# Combining exploratory scenarios and participatory backcasting: using an agent-based model in participatory policy design for a multi-functional landscape

**DOI:** 10.1007/s10980-012-9730-7

**Published:** 2012-03-20

**Authors:** Derek B. Van Berkel, Peter H. Verburg

**Affiliations:** Institute for Environmental Studies (IVM) and Amsterdam Global Change Institute, VU University, De Boelelaan 1087, 1081 HV Amsterdam, The Netherlands

**Keywords:** Multifunctional landscape, Agent-based models, Backcasting, Forecasting, Ecosystem services, Rural development, Landscape evolution

## Abstract

While the merits of local participatory policy design are widely recognised, limited use is made of model-based scenario results to inform such stakeholder involvement. In this paper we present the findings of a study using an agent based model to help stakeholders consider, discuss and incorporate spatial and temporal processes in a backcasting exercise for rural development. The study is carried out in the Dutch region called the Achterhoek. Region-specific scenarios were constructed based on interviews with local experts. The scenarios are simulated in an agent based model incorporating rural residents and farmer characteristics, the environment and different policy interventions for realistic projection of landscape evolution. Results of the model simulations were presented to stakeholders representing different rural sectors at a workshop. The results indicate that illustration of the spatial configuration of landscape changes is appreciated by stakeholders. Testing stakeholders’ solutions by way of model simulations revealed that the effectiveness of local interventions is strongly related to exogenous processes such as market competition and endogenous processes like local willingness to engage in multifunctional activities. The integration of multi-agent modelling and participatory backcasting is effective as it offers a possibility to initiate discussion between experts and stakeholders bringing together different expertise.

**Electronic supplementary material:**

The online version of this article (doi:10.1007/s10980-012-9730-7) contains supplementary material, which is available to authorized users.

## Introduction

Rural areas have long been recognised for their multifunctional character ‘supplying’ the goods and services that sustain human societies (MA, 2005). However, disturbance of natural systems due to intensive agricultural production, alteration of cultural landscapes and land abandonment highlight problematic trajectories for rural environmental sustainability and social functioning. The prevention of these developments has become increasingly linked to effective management of human and natural resources at local scales (Van der Ploeg et al. 2000; Marsden and Sonnino 2008; Wilson 2010). Yet despite this, there has been little investigation of tools that help communities plan and manage local assets for gaining the most benefit from their multifunctional provisioning while maintaining natural capital (O’Farrell and Anderson 2010).

An important attraction of ‘bottom up’ participatory planning design is the ability to integrate local perspectives into development strategies (Pinto-Correia et al. 2006; Stenseke 2009; Shucksmith 2010). Development planning may become more efficient and effective through inclusion of local knowledge for increased sensitivity to place-specific conditions including social conventions, landscape character and environmental characteristics (Tress and Tress 2003; Soliva 2007; Zoppi and Lai 2011). Often participation establishes local legitimacy as stakeholder involvement gives a sense of community ownership (Sheppard 2005; Shearer 2005). Yet, there is also wide agreement that for local plans to be effective different processes occurring at different spatial, temporal, jurisdictional and management scales must be taken into consideration (Cash et al. 2006; Biggs et al. 2007). Complexity originates from different societal demands, rural actors’ decisions, policy and institutional settings and environmental capacities that determine the feasibility of wished developments. Knowledge of these spatial and temporal processes is an important part of understanding regional trajectories and, therefore, for formulating sound interventions in the face of problematic trends (Wilson 2010).

One typical approach to include local stakeholder knowledge is backcasting. Backcasting is a scenario technique where normative targets or unwanted outcomes are defined by a group for the purpose of formulating ways in which such goals can be achieved or avoided (Robinson 2003; Carlsson-Kanyama et al. 2008; Van Asselt et al. 2010; Quist et al. 2011; Kok et al. 2011). The focus is placed on possible solutions to current and future problems rather than prediction of future events. Backcasting can give direction and integrate stakeholders in development planning formulation. One drawback of backcasting is that it may not account for ongoing regional change driven by exogenous processes (Kok et al. 2011). To account for such processes forecasting scenarios can be used, either developed by stakeholders or based on model simulations (Nakicenovic et al. 2000). Model simulations that simplify exogenous and endogenous processes are often used to forecast future trends and help inform about driving factors of development. They have been effective in elucidating the underlying drivers of land use changes (Verburg et al. 2008) aided in the *ex ante* testing of rural policy options (Kathrin et al. 2011) and been revealing regarding problematic development trajectories (Volkery et al. 2008). Although forecasting and backcasting approaches have strong complementarities, there have been few examples where they are used together despite recognition that such integration can help in the effective co-production of development plans (Robinson 2003; List 2004).

This study explores how backcasting and forecasting approaches can serve complimentary roles in participatory development planning. We address two main research questions in reporting our case study experience:

- Are models useful for improving participatory backcasting formulations in stakeholders workshops; and
- What insight can be gained in using forecasting models to test solutions derived from backcasting exercises?

Encouraging open dialogue about model results and using the results in a participatory backcasting exercise is believed to create conditions that stimulate discussion between scientists, decision makers and local stakeholders about rural development planning. By simulating stakeholder suggestions for local intervention, formulated in backcasting exercises, an evaluation of the ideas and strategies for regional development can be made.

## Materials and methods

### Description of the case study region

The study was carried out in the Dutch rural region of the Achterhoek where policymakers and NGOs are seeking to effectively utilize the region’s multifunctional character for rural development. The presence of a unique cultural-landscape is seen as a tourism development asset and unique agri-environmental habitat, which has motivated the introduction of measures for its preservation. However, an ageing farmer population combined with decreasing numbers of farmers and simultaneous intensification of agricultural production may threaten multifunctionality by hindering other rural functions.

**Fig. 1:**
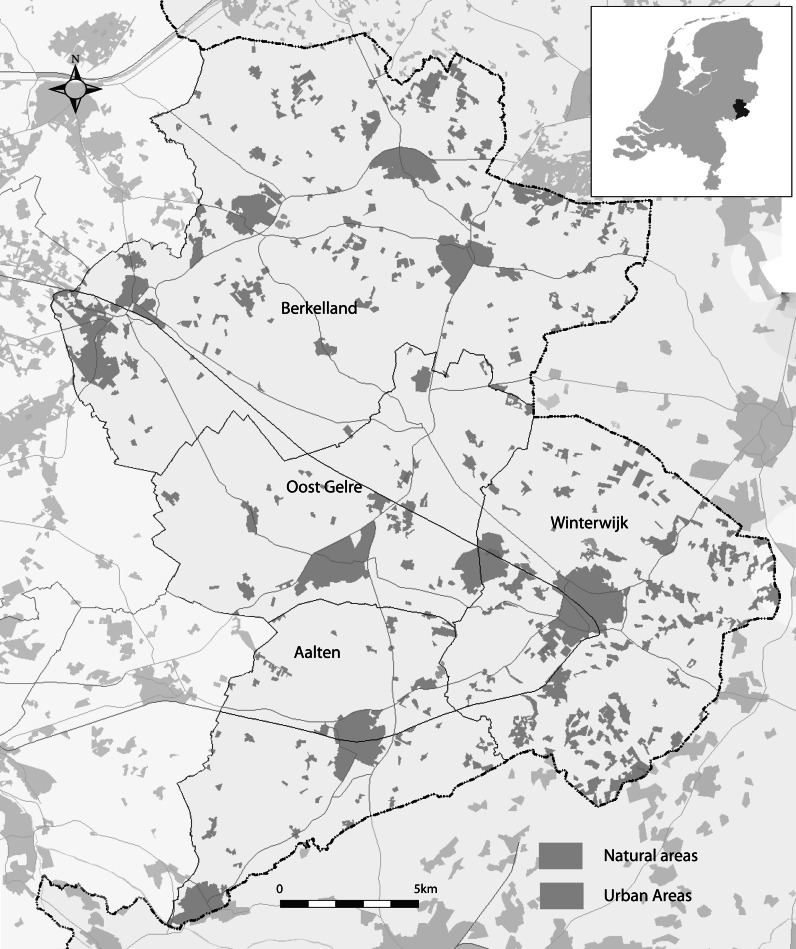
Map of the study area

The Achterhoek is an agriculturally dominated region, located in the eastern part of the Netherlands, which has retained much of its pre-industrial landscape (Fig. 1). This so-called *coulissen* landscape (*bocage*) is characterized by interlinking hedgerows, small agricultural plots and historical farm settlement patterns (Wildenbeest 1989). It is valued for it aesthetic beauty and cultural significance. In part this has contributed to the region’s tourism appeal with an estimated 3.4 million day-trips and 3.7 million overnight stays annually (CBS 2007). However, the cultural landscape also hinders agriculture productivity. Features like hedgerows and tree lines create shadows decreasing production while narrow fields inhibit movement of modern machinery (Wildenbeest 1989; Bont C et al. 2007). A number of reallotment projects have improved agricultural conditions in some areas. Still, local government authorities are concerned that CAP reforms that reduce direct agricultural production payments will result in large farm cessation in unfavourable areas while stimulating intensification in those that are more productive. Increasing numbers of rural residents not primarily engaged in agriculture (>27% of rural population) will also play a larger role in the future of the region (CBS, 2008). Empirical evidence suggests that while they own a small proportion of rural areas, their impact on the landscape is high due to their large numbers and tendency for landscape alteration (Kristensen 2003; Præsholm et al. 2006; Pinto-Correia et al. 2006).

Planners and authorities are exploring options for regional development that retain the unique landscape with the help of EU rural development funds (PG 2007; Polman and Slangen 2008). A policy pilot area, located in the Municipality of Winterwijk, has been established giving land managers (farmers) subsidies for maintaining the cultural landscape (Dienst Landelijk Gebied 2010). It is believed that tourism will be enhanced and biodiversity improved resulting in higher incomes and quality of life for rural residents.

### Methodology overview

**Fig. 2:**
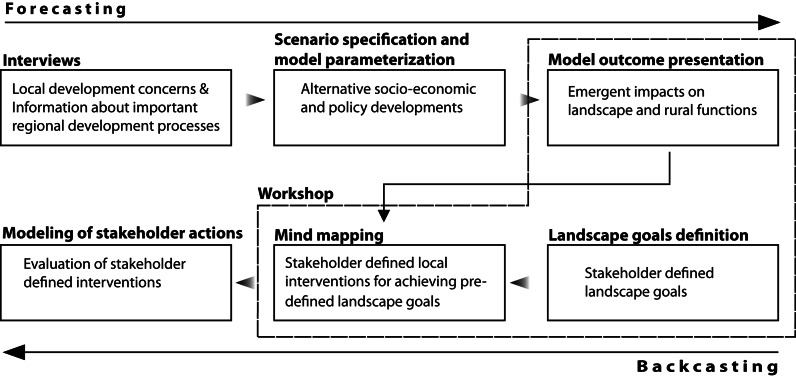
Conceptual model of integrated backcasting and exploratory scenario methodology

At the start of the research, scenarios were defined with the help of local experts and data of current regional trends and development processes. An Agent Based Model (ABM) was constructed to simulate policy scenarios relevant to local stakeholders’ concerns. A stakeholder workshop was held to discuss challenges for different regional developments given the emergent trends depicted in model simulation. Local interventions were jointly defined that could be used to achieve the desired landscape services for the future. Finally, workshop ideas for interventions were added to the model framework to test how they could alter current trajectories. Figure 2 gives the sequences of the research depicting both forecasting and backcasting elements.

### Interviews and scenario specification

New exploratory scenarios for a period of 25 years in the future were defined to address stakeholders’ concern about CAP policy reforms. The majority of respondents were concerned that market liberalisation, currently being considered by the European commission, would drastically alter traditional landscapes and social function in the region. In contrast, respondents felt that payments for landscape service (i.e., maintenance of hedgerows, cultural features and tree lines) and subsidies for small farmers would improve local functioning by attracting more tourism and sustaining the social fabric of the region. Therefore, scenarios that reflect two opposing policy and subsidy options for the case study region were chosen: (i) *More balanced, targeted and sustainable support* (BTS); and (ii) *Abolishment of market and income support* (AMIS). A description is given for each scenario.

#### Balanced, targeted and sustainable support

The scenario BTS outlines reforms aimed at balancing the economic, environmental and social dimensions of rural areas for creating or maintaining synergies between these domains (European Commission 2010). Several reforms to the direct payments scheme are proposed that affect the case study in a number of different ways. A basic flat rate subsidy for all famers would be established. This results in less pressure for small farmers and non-expansionists to increase production through farm expansion. However, the basic rate cap also results in decreased income for both milk producers and large farms leading to fewer resources for production expansion (De Bont et al. 2006). A small-farm subsidy leads to a lesser chance that small farms (farms <10 DSU) will sell their holdings due to favourable earning possibilities. Compulsory aid for the provision of ‘green’ public goods results in a decreased probability that landscape elements will be cut in protection zones (habitat directive areas). In these same zones incentives for landscape elements, such as hedgerows and tree lines, will increase planting or restoration of such features. Furthermore, a focus on rural development will increase subsides for rural residents wishing to diversify. These subsidies are targeted to Local Action Group zones where the LEADER programme is active. LEADER is an EU sponsored programme where farmers receive technical and financial support for (i) the use of new know-how and new technologies; and (ii) best use of natural and cultural resources. The resulting increases in landscape aesthetics leads to increased tourist demand.

#### Abolished market and income support

The scenario AMIS moves away from income support; instead focusing on a limited amount of environmental and climate objectives (European Commission 2010). The European Commission predicts that such a policy scenario would lead to a significant reduction in production levels, farm income and number of farmers; as well as, increases in land abandonment and production intensification. Farming businesses are sensitive to environmental conditions, selling off or abandoning non-competitive parcels. Productive parcels are purchased for farm expansion given their competitive advantage. The phasing out of all direct payment results in a production price–cost squeeze for all farmers, forcing small farmers to either increase production size or sell their land to expansionists. Cross-compliance subsidies are expected to result in the maintenance of special landscape areas only, with nature organisations buying up ecologically significant locations. To increase productivity many farmers choose to cut their hedgerows and tree lines increasing heavy equipment accessibility and reducing tree shadows.

### Model parameterization

In this study, an Agent Based Model (ABM) is employed to simulate possible changes in the landscape for the coming 25 year period (2005–2030). The modeling technique is chosen as it is able to represent local human decisions, institutional settings and the environment, which is not possible with mechanistic large-scale models (Axelrod 1997). This representation of local nuance is assumed to increase stakeholder acceptance of outcome, which is often a criterion for successful knowledge transfer (Sheppard 2005; Shearer 2005). ABM systemise the behaviour of different actors based on their personal characteristics (life stage, management type), location (environmental conditions, other actors) and reaction to different policy changes (Voinov and Bousquet 2010; Valbuena et al. 2010). Agents act independently in an approximation of real world conditions having the ability to interact with other actors through learning and cooperation. Their choices and decisions result in changes to the landscape over time. For this reason ABMs have also been widely used for policy analysis (Valbuena et al. 2010; Kathrin et al. 2011) and in participatory modeling exercises (Guyot and Honiden 2006; Becu et al. 2008). Yet, there have been few examples of ABMs used in decision support (Lempert 2002; Matthews et al. 2007).

**Fig. 3:**
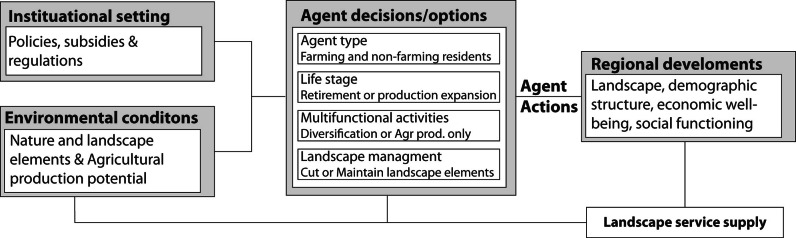
Conceptual framework representing the interaction between policy, environmental, demand and rural actors for simulating regional processes

A model framework developed by Valbuena et al. (2010) was used. The original model simulates farmers’ decisions regarding production expansion, retirement and landscape management. Landscape structure and composition were simulated based on the farmers’ and rural residents’ land choices. Agents’ willingness, abilities and decisions are parameterised based on actual characteristics of rural residents (Jongeneel et al. 2008) and georeferenced according to land holdings. This allows for spatial accuracy of the simulated regional trends (see Valbuena et al. 2008 for a detailed description). A conceptualisation of the model is provided in Fig. 3. Policy and environmental conditions influence the decisions that agents make. Agents’ characteristics including their management type, life-stage (age), multifunctional activities and landscape management preference influence their options and decisions. Their actions result in different regional developments, which influence the supply of landscape services. Each year farmers (agents) decide whether to expand, contract or sell their business to other famers or rural residents. In the same step agents decide whether to retire or continue their farming activities and if they will cut, keep or plant landscape elements.

Farmer decisions are parameterized according to the scenario assumptions (see supplementary material). For example, in the BTS scenario, multifunctional farmers benefit from landscape and nature management subsidies in landscape protection zones, which results in less farm cessation. Farm cessation is further determined by management type and age. Farm expansion is simulated in a similar way. For instance, expansionist farmers in the AMIS scenario are more likely to increase production in areas with few policies restricting intensive production practices (landscape protection). The probability for protecting and planting landscape elements increases in the BTS scenario for all management types. In the BTS scenario landscape management subsidies increases the chance for planting landscape elements. Cutting is probable in locations where there are no restrictions.

To be able to implement the defined scenarios and account for the information obtained during the interviews a few modifications were made to the model. The modifications are based on information provided by local experts and updated policy and demographic projections. Steep farmer population decline (CBS 2010) is incorporated in the model with younger retirement ages and an aggressive land market. Increasing demand for rural estate housing is included by increasing the probability of small aesthetically appealing estates being purchased by urban migrants. Local experts also pointed out that rural residents, hobbyfarmers and retiring farmers have distinctly different land management practices and this is now included in the model. New development zone planning has also been incorporated. Regions earmarked for agricultural development receive an increased probability for farm scale production enlargement, while nature development and wildlife corridors (habitat directive) have lower probabilities. Spontaneous development of these zones by farmers has also been included approximating the observation that diverse farmer types engage in nature stewardship. A further detailed description of the model can be found in supplementary material following the ODD framework for documenting agent-based models as introduced by Grimm et al. (2006).

To check model modifications for stability a sensitivity analysis was conducted (*n* = 50) for each of the scenario runs. Key model parameters were varied to analyse the sensitivity of resulting regional demographics, land use and amount of nature and cultural elements (see supplementary material).

### Stakeholder workshop

A one day workshop was held in the Municipality of Winterswijk with participants chosen from interview respondents, suggestions made by regional contacts and snowballing. The 13 stakeholders that attended represented different policy and planning domains (i.e., water board, local spatial planers, and rural development authorities) and regional expertise in different local sectors (i.e., famer cooperatives and nature and development NGOs). We define stakeholders as those actors who are directly or indirectly affected by an issue, and who could affect the outcome of a decision making process regarding that issue, or are affected by it (World Bank 1996). Due to the importance of overlapping governmental bodies in the Netherlands, care was taken to represent different vertical and horizontal administrative levels, with local regional provincial and national representatives. While care was taken in the selection of stakeholders, scheduling conflicts and interest level limited our flexibility in dictating stakeholder composition. Still, a broad range of perspectives was represented in the workshop with different age categories and genders. Workshop discussions were video recorded for later consultation. One facilitator directed workshop proceeding while three others helped with group exercises.

#### Landscape goal definition

**Fig. 4:**
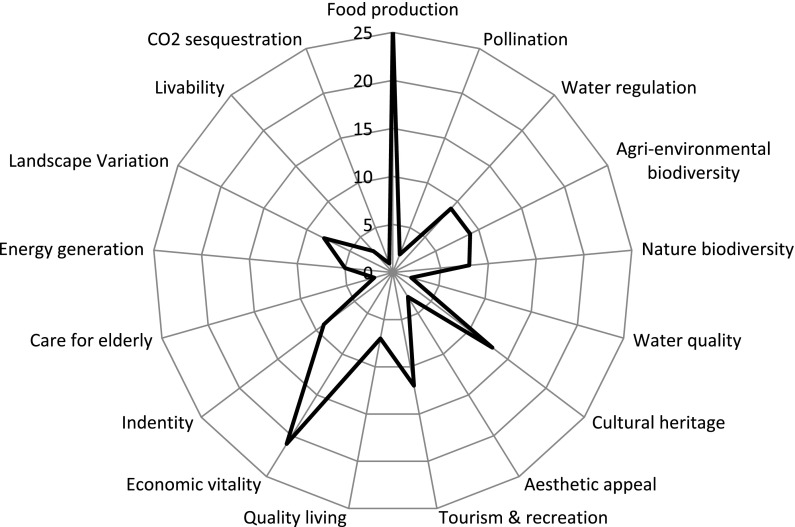
Combined group valution of future landscape service

Workshop proceeding began with an exercise to determine stakeholders’ goals for future landscape and functioning in the region, which is a common methodology procedure in backcasting techniques (Robinson 2003; Van Asselt et al. 2010). Stakeholders were shown a list of landscape services on a poster and asked to allocate ten stickers to indicate how important they perceived them to be (Fig. 4). They were also allowed to add a service if they felt the list was incomplete. Participants were free to allocate all stickers to one or two services or show a more multifunctional ambition by allocating their stickers across the different services. This was followed by addressing some individual answers, which gave the opportunity for clarification of the different interests represented.

#### Model outcome presentation

**Fig. 5:**
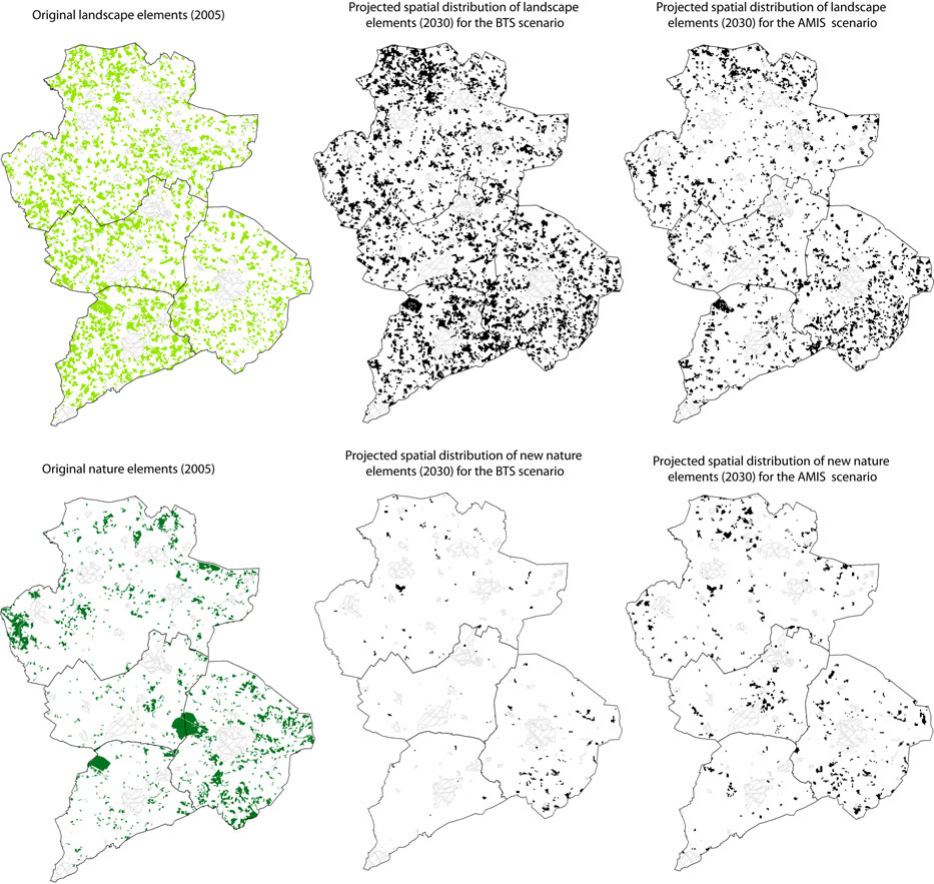
Model simulations of landscape change possibilities for the year 2030

The stakeholders were then presented the results of the model simulations. These model results depicted different landscape outcomes for the two policy scenarios from 2005 to 2030. It was believed that this would further frame and inform workshop participants’ understanding about the feasibility of their goals given the temporal and spatial projections of rural actors’ (farmers, rural resident) landscape choices. Model results were presented to the group using a number of different indicators including demographic change and structure, availability of economic opportunities, and environmental conditions (Table 1). Maps depicting changes in landscape elements and nature for the two scenarios were presented to stakeholders, highlighting and comparing a number of spatial temporal changes (Fig. 5). These maps were also overlain with current wildlife habitat ranges and popular tourist sites to indicate possible future landscape service trade-offs. Specific attention was paid to explaining the causality between the ongoing socio-economic processes and the simulated landscape changes to achieve an understanding of the challenges faced by the region. Participants could visually compare and react to the presented results.

**Table 1:** Simulated indicators of quality multifunctionality

	BTS			AMIS		
	2005	Simulated	Change	2005	Simulated	Change
Total number of farmers	1705	1230	−475	1705	1204	−501
Average farm size (ha)	14	31	17	14	31	17
Total agricultural area (ha)	45765	45254	−511	45765	44075	−1690
Percentage of multifunctional/diversified farmers	31	16	−15	31	16	−15
Percentage of rural resident not primarily engaged in Agri.	38	40	2	38	40	2
Percentage change in the length of Landscape elements			+24			−20
Semi-natural areas (ha)	5045	5612	567	5045	6915	1870
Average distance to farthest parcel of land (km)	15	19	4	15	19	4

#### Mind mapping

After the presentation the stakeholders were split up into three subgroups with the goal to formulate actions to be taken in order to achieve wished rural functionality while accounting for the ongoing developments simulated by the model. The exercise was carried out using mind maps. Cognitive or mind maps are widely used in workshop settings to structure and systematize group understanding of key concepts and/or issues (Soini 2001; Evrekli et al. 2009; Kok et al. 2011). The groups were given poster paper, markers and Post-Its^™^ and asked to formulate their ideas for achieving the predefined landscape services goal. A facilitator helped organise stakeholders’ ideas by printing discussion points on the paper and aided in action formulation by way of verbal prompts and inquiries. Often in backcasting exercises the goal is to limit researcher influence on stakeholder developed outcomes to induce creativity and ensure stakeholder representation (van Vliet et al. 2010; Kok et al. 2011). However, in this technique we prompt participants with information based on model projections. The mind mapping exercise allowed us to ascertain if stakeholders’ accounted for the endogenous processes depicted in the model.

Each stakeholder group was then asked to present their mind maps, followed by a discussion about the different suggestions. It was also explained that the suggested intervention would be evaluated by way of model simulation after the workshop. This would offer the stakeholders insights regarding how local interventions would influence regional outcomes. At the culmination of the workshop a questionnaire was administered testing workshop satisfaction, the perceived utility of the different techniques employed and perceptions about different policy options for the development of the region.

### Modeling of interventions suggested by stakeholders

**Fig. 6:**
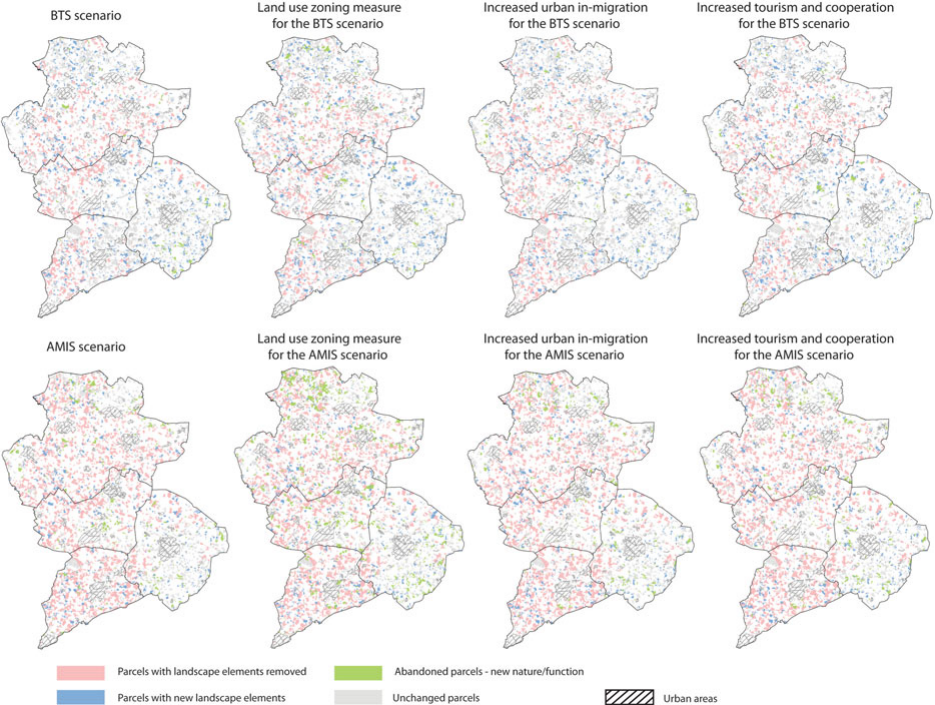
Model simulation of projected nature and landscape element changes (2005–2030) with stakeholder groups’ interventions suggestions

Three proposed policy interventions based on the workshop outcomes were added to the model to evaluate their effectiveness. The policy interventions are simulated by adding agent rules, varying the intensity of key variables (e.g., constraint limits) and including a sub model to approximate a stakeholder observed actor interaction. The different simulated scenario results were then compared (i.e., demographic changes, economic opportunities, and environmental condition). Maps of the resulting landscape evolution were also compared with the original projected changes (Fig. 6). The use of model simulations for testing local interventions is increasingly been seen as a way to aid in policy makers deliberation about implementation (Pannell 1997; Lempert 2002; Matthews et al. 2007). For this reason a detail report explaining the result of the simulation outcomes was made and sent to workshop participants for their evaluation and information.

## Results

### Interviews and model parameterization

Interview responses revealed considerable local understanding of regional development processes. Respondents discussed how the historical evolution and environmental condition had influenced current landscape patterns. This was linked to the cultural-heritage function, agricultural production conditions and the provision of important ecosystem services like water quality and wildlife habitat. Appreciation and understanding of these local processes indicated strong local governance capacity. This was confirmed by respondents who explained that strategic rural development meetings between policymakers, municipal planners, NGOs, agricultural cooperatives and academic institutes were frequent. Respondents had similar ideas about regional challenges and how best to tackle problematic developments. They cited the aging farm population, increasing agriculture production intensification and depopulation as areas of concern. Furthermore, decreases in farm subsidies was thought to increase the vulnerability of small farm businesses and expected to interfere with the identity and character of the region. To represent these developments the Abolished Market Support (AMIS) scenario was developed. A majority of the respondents felt that payments for public goods were an important part of maintaining the landscape and therefore increasing the development opportunities for the Achterhoek. For this reason a scenario with payments for ecosystem services and small farm protection was developed (BTS). Respondents also told about increasing numbers of urban residents purchasing small farms in the region, and this was added to the model.

### Model simulation results

The model simulations of the two scenarios revealed distinctive differences in landscape evolution, but, surprisingly, little difference between the projected socio-economic indicators.

In both scenarios similar farmer population decline was apparent suggesting an emergent trend of decreasing social function through depopulation (Table 1). A decrease in the proportion of diversified and conventional farmers in comparison to expansionist indicates a decreasing number of the smaller production systems types. Increases in average farm size in both scenarios show concurrent processes of farm expansion, which is persistent despite subsidies for small-farms and a direct payment cap in the BTS scenario. This is driven by large proportions of farm expansionists that due to their market orientation are similarly successful in both simulations. The similarity of simulation outcomes between the two policy scenarios shows that different policy interventions may not substantially influence these socio-demographic pressures (Jongeneel et al. 2008; Wilson 2010).

Although similar changes in socio-demographics occur in both scenarios, simulations did indicate substantial difference in landscape evolution between the two policy scenarios. In the AMIS scenario there are significant increases in semi-natural area in comparison to current patterns. Landscape protection areas, habitat corridors and wildlife protection zones all experience agricultural abandonment as farmers take these parcels out of production or nature organisations purchase them for nature development (Fig. 5). In the same scenario there are significant decreases in the *coulissen* landscape as farmers choose to increase production efficiency through tree line and hedgerow removal for land consolidation. Figure 5 depicts the spatial distribution of these landscape changes in areas that are earmarked as agricultural development zones and where the landscape has been more significantly rationalised. In the BTS scenario the number of landscape elements increases. Economic incentives for the management of cultural and agri-environmental habitats induce land managers to protect these landscapes and in some cases plant new elements. As a result of landscape management subsidies there is limited agricultural abandonment with farmers choosing to capitalise on the subsidy earning possibilities. The survival of market oriented farmers drives these landscape alterations as they are more prone to economic optimization than diversified farms (Table 1). However, the results also show an increasing number of rural residents with non-agricultural incomes indicating that these actors will be major contributors to future landscape dynamics.

### Workshop

#### Future landscape service definition

The results of the exercise for determining desired landscape services revealed that stakeholders wish to have a mix of functions for the future, with the majority of stakeholders (*n* = 8) spreading their votes equally amongst the proposed landscape services (Fig. 4). However, two distinctive opinions about the role of agriculture for such multifunctionality were apparent. One segment of the workshop participants viewed agriculture (*n* = 9) as key to future rural functionality while another saw nature services and high quality living as more important (*n* = 5). This split was revealed in the group discussion with several respondents advocating less funding for agriculture as a way for encouraging new non-traditional land uses. One participant described their wishes for development illustrating this perspective, “We need to focus less on subsidies for farmers and be open to new and innovative uses of the region”. The other segment cited the maintenance of the agricultural landscape as interrelated with the identity and character of the region thus requiring government support for its retention. This was evident in discussion about alternative functions with a participant expressing concern for conservation of the landscape, “It’s the unique cow breeds and land management that gives this region a rich colour and character…that is why tourist come here, that shouldn’t be lost”. Despite differences in opinion about which functionalities should be pursued for the future, there was agreement between stakeholders that continuation of agricultural functionality while balancing the economic vitality and nature quality of the region was a positive endeavour. Participants verbally agreed with the statement of one participant when saying, “Any development must adhere to the local character of the landscape for it to achieve a benefit for the region”. This finding suggests that while the technique used allows for quantification of the different opinions represented it is also an acceptable way to synthesize the different wishes for further discussion of future planning. Stakeholders’ evaluation of the exercise were split with seven participants agreeing that the technique helped in understanding the different stakeholder perspectives represented in the workshop and seven neither agreeing or disagreeing on the Likert scale.

#### Model outcome presentation

Stakeholders’ evaluation of the usefulness of the model outcomes for better understanding regional processes was mixed. The discussion after the presentation of the model results was interesting and focussed on the causality of the processes underlying the results. However, ten out the thirteen respondents neither agreed nor disagreed that they better understood how CAP policy reform would alter their community, with only three agreeing. A similar result was recorded for understanding how demographic trends would affect their region (4-disagree; 5 agree/disagree; 3-agree) and future implication for the different rural economic sectors (4-disagree; 4-agree/disagree; 4-agree). Nonetheless, respondents did answer that they learned more about the role that different actors play in forming the landscape with seven agreeing that they better understood this endogenous process (1-disagree; 5-agree/disagree). Stakeholders also said that they learned more about the spatial dynamics of the region with nine respondents agreeing that they better understood these processes (3-agree/disagree; 1-disagree).

#### Mind mapping

The mind mapping was better appreciated with 11 respondents finding that the exercise was helpful for bringing structure to group ideas and 11 respondents finding it good for developing solutions to development challenges (2-neither agreeing or disagreeing). It resulted in a number of ideas about actions for achieving the landscape functionality as indicated by the sticker exercise (i.e., continuation of agricultural functionality while balancing the economic vitality and nature quality of the region).

The groups defined similar local development challenges linking these to both endogenous and exogenous pressures. For instance global food competition was linked to homogenization of the landscape through the need for agriculture production intensification. Eutrophication of waterways was likewise related to this market pressure. Abandonment of old farm buildings and loss of traditional landscape was associated with ageing and depopulation. This was further linked to future long-term issues related to the erosion of the local tax base, which would limit the governance capacity. The inclusion of processes shown in the model simulation in all mind maps was a confirmation that stakeholders recognise similar challenges to those depicted in the model.

Many similar suggestions were made between the three groups for local interventions to solve development issues (Table 2). All agreed that intensive farmers should be encouraged to leave locations in sensitive landscapes while encouraging small and multifunctional farming in nature and landscape protection zones. One respondent said this plainly, “We need the right farmers in the right place”. The groups suggested reallotment schemes, zoning restrictions and location specific subsidies for cultural landscape and nature management to achieve this goal. Attracting tourist was also viewed as a positive development. To increase tourist numbers stakeholders suggested maintaining the landscape, which again was interrelated with clustering multifunctional farmers in pre-existing cultural landscape. Public–private partnership was seen as a policy option for creating income diversification opportunities in tourist hot-spots like organic products produced by multifunctional farmers. Many suggestions also focused on making the region more attractive for entrepreneurs and economic investment. An advertisement campaign to promote a competitive image, a programme for investing in or removal of abandoned farm buildings and the installation of high speed internet cable were suggestions made for attracting new people and business. Disagreement occurred with suggestions for increased economic output by way of targeted subsidies for new economic sectors like energy generation. One stakeholder warned that market orientation would result in the homogenisation of the landscape and loss of landscape richness. There was agreement that any development or innovative function would need to adapt to the surroundings of the landscape to maintain the landscape character. For this, cooperation between local government and entrepreneurs and between farmers was agreed to improve the ability to create synergies between economic sectors. For example, offering complimentary tourist activities by neighbouring farmers or cooperating with local policymakers to set up larger diversification projects (community lead initiatives).

**Table 2:** Stakeholder derived policy intervention for the realisation of wished landscape service

Activation of positive process	Local measure interventions
Re-zoning of farm management types to appropriate environmental locations	Land reallotments schemes Restriction and zoning based on landscape profiles (attractiveness, environmental robustness) Nature farming in environmentally sensitive areas Economic valuation and remuneration of nature services Regulate synergies between functions Targeted subsidies for different environmentally appropriate uses Communication between different stakeholders
Attract tourist	Increase cooperation between entrepreneurs and policymakers Maintenance of the landscape (promotion of diversified farms) Organic and local products
Attract entrepreneurs	Invest in local social cohesion Promote the region to outsiders (Advertising campaign) Prevent degradation of landscape aesthetics while allowing for some restructuring to help develop new functions Continual adaption of zoning plans to stay in step with new innovations (e.g. Solar-panels)
Increase economic output/ diversification	Promote new economic sectors through correct economic incentives (e.g., niche markets in organic products) Develop appropriate infrastructure for entrepreneurs (e.g. fibre optics) Targeted subsidies for business types that fit the local character Macro-credit for large projects Landscape restructuring (e.g. empty barn/building schemes) Innovation assistance—smart non-partisan solutions Consider other incentives than subsidies A decentralised communal funds for community lead initiatives
Develop an energy landscape	Create a synergistic cycle where small scale farms produce material from hedgerows, which supply on farms bio-digester giving incentive to maintain the landscape for fuel that in turn attracts tourism

### Simulation experiments

Not all interventions proposed could be simulated given limitation of model functionality and available data. Three possible solutions raised during the mind-mapping exercise were selected: land use zoning, increased tourism demand in conjunction with cooperation between farmers, and increased in-migration. The measures for re-zoning farm management types to appropriate environmental locations was achieved by restricting intensive expansionist farmers from expanding or bequeathing their farms in landscape protection areas, habitat directive areas and cultural landscapes. Instead these actors are required to sell their parcels to multifunctional famers, rural residents not primarily engaged in farming or a nature conservation organisation. The interventions were simulated both in the AMIS and BTS scenarios. The alteration results in sharp declines of intensive agriculturalist in zones where the landscape and nature is highly protected. In Winterswijk, for example, for the AMIS and BTS scenarios there is a 56 and 63% decline in this farm type respectively in comparison to original projections. Figure 6 shows the landscape evolution of the different policy actions simulations in comparison to both the original scenario projections. For the land zoning measure there is increased agriculture abandonment as there are too few multifunctional farmers willing to buy up land in highly regulated zones. This is significant in the AMIS scenario with clusters of agriculture abandonment around protected areas but less pronounced in the BTS scenario. To simulate cooperation and tourism, the model was modified to include stakeholder interactions. Agents assess the management techniques of their ten nearest neighbours, and cooperate with them in diversification activities. Such management strategies are related to increased demand for nature friendly products and tourism observed in the region and elsewhere (Præsholm et al. 2006; Jongeneel et al. 2008; Wilson 2010). Non-multifunctional farms can adopt multifunctional techniques if there are four multifunctional farmers nearby and they are located in an area with tourist assets (Nature, hedgerows, attractions). With 10% cooperation and 10% increase in tourism demand there is a 17% and 8% increase of multifunctional farmers in comparison to the original BTS and AMIS projections without the intervention respectively. The difference in cultural landscape comparing the policy action to the original projections is small. However, in Winterswijk there are fewer landscape changes as multifunctional farm numbers increase and landscape elements are better protected (Fig. 6). A programme to attract urban in-migration was simulated through increasing demand for smaller rural residencies and decreasing requirements for aesthetically pleasing landscapes around the potential housing locations. The procedure did not result in significant difference in numbers of new rural residents in comparison to both scenarios projections despite increasing the probability of purchase to 100%. The availability of small farms determines the number of urban migrants settling in the region. Still, there is a clustering of rural residents not primarily engaged in farming in aesthetically pleasing areas resulting in fewer changes to the landscape in comparison to original projections (Fig. 6). Interventions are in general less effective in the AMIS scenario as land abandonment increases or a monofunctional agricultural landscape is developed. Market competition leads rural land managers to adhere to market pressures more than local intervention in this case.

## Discussion and conclusions

### The role of exploratory scenarios in backcasting

In this article we explored the possibility of employing an ABM to support stakeholder discussion and a backcasting exercise. The results of the stakeholder process were evaluated with the same model. Often model and stakeholder-based assessments are disconnected and separate activities. Examples of approaches that integrate stakeholder and model based techniques include the joint definition of scenarios with stakeholders that are modelled afterwards (Etienne et al. 2003) or role playing games where agents assume different roles from which model parameters can be tested or collected (Voinov and Bousquet 2010). Model results are then used to explore and discuss likely challenges emerging from alternative future events. Unlike these approaches, stakeholder participation in this paper is achieved by way of goal and solutions formulation placing emphasis on supporting stakeholder deliberation of sound development strategies. The backcasting enables examining goals for the future in the context of developing trends simulated by the model (Potschin et al. 2010). Discussion between experts and stakeholders helped in assessing the desirability of future outcomes while bringing together different expertise and knowledge of how desirable outcomes can be achieved (Robinson 2003). As the successful development or maintenance of multifunctionality relies on understanding and anticipation of complex processes and local reaction to these processes such novel approaches will be increasingly required if rural communities are to be able to gain wider benefits from their multifunctional provisioning.

The results indicate that model forecasts helped stakeholders to formulate rural development ideas that incorporate aspects of endogenous, spatial and temporal processes affecting their region. This was evident by the acceptance of model outcomes and by the inclusion and discussion of these processes by stakeholder groups in the backcasting exercise. While the model was appreciated for illustrating the spatial dimension of issues affecting rural development, the policy changes that were addressed were less provocative for stakeholders. This is likely due to the translation of abstract processes already understood by stakeholders like policy reforms and demographics change into concrete spatially explicit illustrations. When asked if the workshop added to the current debate about development planning, several of the participants agreed citing the novelty of using the models. One participant summed up this group appreciation saying, “The model shows [in the maps] what we were concerned about explicitly; we thought that market liberalisation would be problematic for the cultural landscape and that was the result”. Likewise, participants were pleased with the inclusion of different management types, with many recognizing the importance of the spatial heterogeneity of different decision-making actors for the landscape. In group discussion participants were interested in the make-up of their particular municipality and made inquiries regarding how one management type was defined in relationship to the others. They gave examples of their experiences with different actors that fit, and in a few cases did not fit, with the management type characterisations used in the model. Stakeholders’ suggestions for restricting intensive farmers from sensitive environmental zones is evidence that spatial issues were considered and related to management types. The use of an ABM model allowed for the inclusion of these different management types.

Testing different proposed policy actions through model simulations likewise can further help decision makers and stakeholders understand the implication of interventions beforehand. For instance, the model outcomes demonstrate that the promotion of in-migration will require a stock of housing that is suitable for urban migrants to purchase. Zoning policy must also consider the willingness of farmers to engage in certain management styles, as was illustrated by increased agriculture abandonment with the intervention. Intervention can also have distinct spatial consequences where zoning can marginalise certain activities (intensive production) and valorize others (multifunctional). This can result in a clustering of different land uses increasing intensification, whether that is tourism or agriculture. Comparison of interventions across the two scenarios indicates that endogenous economic processes influence the effectiveness of local policy interventions to improve socio-economic conditions at the local scale. Local intervention may be nullified with increasing market competition as farmers are motivated by production efficiency.

### Models in a joint-learning process

The issue of knowledge transfer and learning effects has been highly debated in both scenario development and modeling literature (Vervoort et al. 2010; Lagabrielle et al. 2010; Pettit et al. 2011). The result of the questionnaire and discussion, however, did not unequivocally demonstrate a learning effect (Fig. 4). While it is often ubiquitously stated as an advantage of participation, these findings suggest that learning is particular to each stakeholder’s understanding of local processes (Sheppard 2005) as stakeholders were largely aware of demographic and policy change challenges. Given that there was no ‘zero-measurement’, where the learning outcomes without the use of the model can be compared to, it is difficult to gauge to what extent model outcomes improved the mind-mapping exercise. Beyond learning, the goal of the approach was to focus stakeholder discussions and structure the mind-map exercise, which was agreed to be the case by the participants.

The perceived legitimacy of model outcomes by stakeholders in model-aided decision support is widely recognised as a requirement for the success of learning and solution development. If stakeholders feel that model results are not adequate or incorrect, the participatory process can grind to a halt (Lagabrielle et al. 2010). Often this can occur when stakeholders are not involved in the modelling process (Voinov and Bousquet 2010). In our study, stakeholders expressed confidence in the model output during the workshop. The inclusion of local expert knowledge about local processes helped in creating this legitimacy, as processes and actors well known by local stakeholders to influence regional development were included. However, the creation of model credibility may have led to the situation where stakeholders were not forced to ‘think outside the box’ regarding alternative trajectories, regional challenges and policy action solutions (Xiang and Clarke 2003; Vervoort et al. 2010). This was evident with many similar suggestions made by the different groups in the mind mapping exercise. Still stakeholders were well aware of model limitations questioning model validity and suggesting that air photos, from the past and present could be used to increase the credibility of projected results.

### Participatory policy design in practice

In this study we demonstrate a method of participatory policy design that could be used in practice. While the single case limits the wider applicability of our findings for policy design, several practical lessons can be drawn from our experience. The experience of the workshop led to the realisation that terms used for presenting model findings and in stakeholder exercises needs to be understandable and relevant to stakeholders. Stakeholders found the terminology characterising the landscape services in the sticker exercise ambiguous and incomplete, which may have contributed to the poor assessment of the technique in the questionnaire. Still it did activate a rich debate about what constitutes a landscape service and how such provision could be harnessed for regional development. Two key alterations can be suggested for increasing stakeholder appreciation (a) terminology may be simplified and oriented toward local planning and decision discourses; and (b) emphasis can be placed on the synthesis forming aspect of the exercise. Such an approach could be used in backcasting exercises when time constraints prevent drawn-out group deliberation for goal definition (Kok et al. 2011).

The use of maps and visuals to enhance stakeholder discussions in participatory decision support has been growing in the last decades with the acknowledgement that spatial representation can aid in finding solutions that are appropriate to location-specific conditions (van Berkel and Verburg 2011; Arciniegas et al. 2011). In our study, stakeholders were required to visually compare regional maps depicting scenario outcomes for the better understanding of regional development. Empirical evidence suggests that stakeholders often find it difficult to think in spatial terms preferring instead an issue-based discussion (Etienne et al. 2003; Lagabrielle et al. 2010; Pettit et al. 2011). This raises the question: how important are spatial representations for stakeholder dialogues? The findings in this study demonstrate that landscape processes including variation, structure and function are important to understand when considering development and that stakeholder appreciate the description of them in model visualisations.

In the Dutch context, local policymakers are often required and/or frequently requested to join different (science-policy) workshops as stakeholders of their policy field. This is especially the case in the study region where a multitude of workshops have been conducted over the past years. Repeated interaction with nature organisations, scientists and other policy bodies in these exercises can stimulate innovation, but also result in a situation where workshops become a routine for participants. Combating apathy caused by common workshop procedures and results is an important consideration in workshop design. Packaging model results within alternative formats of interactive workshop exercises is one way to prevent workshops from becoming mundane.

One noteworthy benefits of using such methods for increasing stakeholder participation is that it helped to clarify the different opinions held by the participants regarding alternative development options and solutions (Valkering et al. 2010; van Berkel and Verburg 2011). The sticker exercise gave a picture of different values represented at the workshop. Such inventory is often overlooked in participatory exercises, while still recognised as an important aspect of overall workshop outcomes (Soliva 2007; Metzger et al. 2010). Individual sticker allocation helped in distinguishing two groups of stakeholders, giving context to the suggestions made in the mind map sessions, and offering insight into the different perspectives regarding regional development.

This is an important feature of such participatory method as often there are competing and conflicting interests for development, which was evident in the workshop. Although there was agreement between different policy and planning stakeholders that a multiple function strategy should be pursued, this did not translate into consensus about in which form and how to achieve this. Participatory exercises where different perspectives are represented, like the tools demonstrated here, can help clarify the differences and similarities about future development wishes. The exercise shows that there are tradeoffs, both between different functions but also between different stakeholder groups.

## Conclusions

Increasing decentralisation of decision-making in many EU countries invariably means that local decision-makers will become more involved in formulating local interventions (Shucksmith 2009). Investing in local capacity for thinking long-term about landscape, demographic and policy evolution can help in the identification of problematic trajectories for multifunctional provisioning. To aid stakeholder participation and provide well-informed discussions innovative tools are needed to structure decisions about complex issues such as landscape functionality. Decision about future functionality will include multiple trade-offs between functions, spatial and temporal scales and different stakeholders. This paper has shown that participatory methods can integrate tools like an agent based model by helping anticipate locations where emergent changes can occur and testing different ways to alleviate identified problems. From this understanding intervention can be tailored to specific management types and geographic locations that are efficient in providing the desired functionality.

## Electronic supplementary material

Below is the link to the electronic supplementary material.

Supplementary material 1 (DOCX 22 kb)
