# Supplementary material for: Combining exploratory scenarios and participatory backcasting: using an agent-based model in participatory policy design for a multi-functional landscape
Source: Landsc Ecol. 2012 Mar 20;27(5):641–58. doi: 10.1007/s10980-012-9730-7 (PMC4426888; doi:10.1007/s10980-012-9730-7)
Supplement: Supplementary file 1 — Supplementary material 1 (DOCX 22 kb) [file 10980_2012_9730_MOESM1_ESM.docx]

**Supplementary material -** **ODD Model description after Grimm et al. (2006)**

*Overview*

The model framework was parameterized in NetLogo 4.1. The simulation consisted of 20 steps or years and is based on the model developed by Valbuena et al. (2010). This overview describes amendments to the original model as details can be found in the aforementioned publication.

*Purpose of the model*

The objective of the model was to simulate the evolution of landscape processes related to policy and demographic trends for communicating possible emergent development issues to planning and policymaking stakeholders. To achieve stakeholder buy-in and test stakeholder derived suggestions one decision-making processes and four sub processes were added to the original model framework. These are: farm diversification, a tourism demand influence, spontaneous land stewardship, zoning regulations for different land uses and a policy rupture.

*Entities, state variables and scale*

Agents represent farmers (farms) and rural residents not primarily engaged in farming (estates) each with different characteristics (i.e. life-stage, multifunctional activities and landscape management preference). Rural residents and farmers are distinctly characterized as they are understood to have different decision making patterns and options. For example, farmers are characterized with an agri-business type, production scale and memory of their past land decisions (5 years). This influences their decisions and options in relation to policy, subsidies and regulations; as well as, environmental conditions. Rural residents are less influenced by the institutional setting as they are not engaged in farming activities. Their decisions and options are based on lifestyle and aesthetic characteristics. Environmental conditions like the presence of landscape elements and nature determine where they will seek to live. A number of policy zones and area characterizing the location specific assets were included. Agents located in these areas utilise different subsidies and/or be restricted to certain actions. This includes landscape and development initiative areas (Local Area Groups – LEADER scheme, spatial planning and landscape subsidy zones) and locations with tourism attractions (i.e., camping sites, Bed and breakfast, walking and biking trails, castles, organic and nature farm visits). Distance to like management types is also calculated representing the ability for agents to cooperate with one another.

*Design concepts*

The emergence in this model, like the original model, is represented primarily in changes to landscape structure. Adaptation is included in actors’ interaction with agents whereby each evaluates their neighbours’ management type in deciding whether to adopt similar practices. This is influenced by tourism demand in locations where there are tourism attractions. Tourism demand is determined by the amount of landscape and nature elements in the case study area. The distribution of multifunctional farmers throughout the case study area is assumed to allow for cooperation between different farm types if there is a tourism demand. However, the factors that influence diversification are diverse. Spatial characteristics (Pfeifer et al. 2009) attitudes (Jongeneel et al. 2008) and economic conditions (Meert et al. 2005) are each influential factors that could not be simulated. Observation is done by way of sensitivity analysis and comparison of simulation experiments. Stochasticicity is included in market demand for tourism as this is a highly variable endogenous process. The management type of new farmers is also stochastic.

*Initialisation*

The model is initialized as in the original framework with the exception of the inclusion of a number of new spatial data sets.

*Input data*

Several data sets were added to the original initialisation to enable added functionality and increase accuracy in parameterisation and projections of demographic figures. A new agri-businesses census is used to more accurately depict rural processes observed in the Netherlands with higher concentration of farmers 65 and older retaining the majority of small farms (CBS 2010). Modelled data has also been drawn upon that simulates survival of agribusiness types in different policy scenarios where farm aid was capped (De Bont et al. 2006). The scenarios tested are based on policy options currently being considered by the EU. Cadastral data was obtained from provincial spatial planning databases (PC, 2010). Agricultural development, habitat directive, corridors and protection of cultural landscapes zones and Local Areas Groups (LEADER) were initialised and used to calculate probabilities for agricultural expansion, hedgerow and tree line management and diversification for each actor. Likewise a tourist asset maps was developed to localize tourism activities contributing to diversified demands.

*Submodels*

Farm cessation, farm expansion, protection of landscape elements and endogenous diversification are submodels that have been added to the model. Each agent’s options, initial circumstances, periodicity and parameters are unique to their actual characteristics. For each simulation experiment agent parameters were altered to adhere to the assumption described in scenarios and policy solutions suggested by stakeholders. Only the alterations to the submodels are described.

The process of farm expansion was augmented to simulate production and farm size decline in farmers older than 65, which was evident in updated national statistics. Approximating demographic/agri-business size ratios is done by decreasing the probability for expansion in farmers older than 65 that own farms larger than 50 dsu by a factor of 10% per step. Farm expansion now also includes provincial planning zones. In the AMIS scenario, expansionists located in zones earmarked for agricultural development have an increased probability (50%) for expansion while non-expansionists are less likely to expand (25%). This is less pronounced in the BTS scenario with 10% increase and 10% less likelihood respectively.

The process of farm cessation has been altered to include the geographical extent of the Local Area Group (LAG) as part of the LEADER scheme. The LEADER programme in the case study area is involved with support for i) the use of new know-how and new technologies; and ii) best use of natural and cultural resources. It is assumed that this results in less farm cessation for diversified/multifunctional farmers. This is approximated in the model by altering the survival rates of different management types. In the AMIS scenario non-expansionist are 50% more likely to stop, while in the BTS scenario diversifiers are 50% less likely to stop. Parameters determining probability for agribusiness type cessation have also been altered. Economic models that closely approximate the farm aid cap proposed by the EU are used (Bont et al. 2007). Dairy farmers are most affected in these modelled outcomes. Their chance of stopping is 10% if a flat rate cap is establish (BTS) and 15% if subsidies are phased-out at the 50 percentile mark (AMIS). The cessation action is also altered by way of an economic/policy rupture. Policy implemented by the EU in 2013 alters earning possibilities, amends potential for expansion due to different restrictions. It also simulates greater opportunity for farm diversification and survival through capacity creation schemes (LEADER) for the different agents. In the AMIS scenario opportunities for expansion is increased to 15% which also increase the chance for farm cessation in all farming agents. In the BTS scenario a 3% increase is applied, which results in less farms cessation and expansion. The AMIS scenario has fewer restrictions for expansion and protection of landscape elements in important habitat areas (habitat directive) in comparison to BTS.

Protection of landscape elements has been altered for rural resident stakeholder only, as empirical evidence and local experts indicated different landscape management for these actors (Kristensen 2003; Præsholm et al. 2006). A 30 % chance of planting landscape elements for all rural resident actors is used. This is a conservative estimate in comparison to empirical findings regarding plans for future landscape alterations for these rural actors (Præsholm et al. 2006). This conservative estimate is adopted as rural residents keep landscape elements already located within their holdings and also have the ability to plant new elements. The option for spontaneous landscape stewardship by conventional farmers is also now included to reflect the comments of local experts that diverse farmer types have adopted the practice of landscape protection in the region. Conventional famer have a 10 – 3%, depending on the scenario, of planting landscape elements.

Endogenous diversification was added to simulate suggestions made by the stakeholders in the workshop about cooperation and tourism. This can be linked to empirical finding of increasing consumption of urban residents of rural products and services for example with bed and breakfasts, farm experience activities and tourism and recreation (Jongeneel et al. 2008). Non-diversified farmers assess the management practices of their 10 nearest neighbours in deciding to diversify. It is assumed that there is a learning effect if he/she is located near four farmers engaged in diversified activities. The decision to diversify is likewise determined by tourist demand. Tourism demand is dependent upon the availability of cultural and nature elements. An increase in these elements and a stochastic outcome results in more tourism demand. Farmer-agents located in a Local Area Group zone are assumed to have an increased chance for diversification (50%), as development funds are available for diversification in these LEADER schemes areas. Endogenous diversification is possible if non-multifunctional farmer cooperate with multifunctional farmers. Their proximity to 4 multifunctional farmers is assumed to create this cooperation. Non-multifunctional farmers decide whether they will engage in more multifunctional activities given this cooperation and surrounding opportunities for tourism (landscape elements, nature and tourist attractions).

Urban migration has also been altered to reflect the findings of the interviews. Expert relayed that urban residents are increasingly moving into the region purchasing small estates with aesthetically pleasing surroundings. To reflect this, small farms (< 10 DSU) can be sold throughout the region. The probability for selling to an urban resident is determined by the presence of nature or landscape (10%) elements in a neighbourhood of 1km (circular) around the homestead.

*Submodel simulation experiments*

The key parameters contributing to model outcomes were varied to test their influence on model outcomes. The influence of the demographic make-up was tested by decreasing the ages of the actors by 5 year increments maxing out at 20 year. For each incremental change the average farm size decreases, with a 20 year downward shift resulting in a 10% decrease in average farm size. Younger farmer ages result in fewer retirees and therefore fewer farms and/or parcels for purchase. This prevents the purchase by individual farmers of more parcels. The large number of retirement age farmers is a major driving factor for farm scale expansion in the region as younger farmers purchase their land. The impact of market forces was tested by controlled increases in the amount of development funds available to land managers for expansion and the number of farmers stopping each year. While, purchasing power had little influence on farm size, small increases in the number of retirees resulted in larger farm sizes. For example augmenting the original cessation rate for all farmers from 0.029 % to 0.05 % in the BTS scenario resulted in a 5% and 6% increase in average farm size for intensive producers and all farms respectively. Again this indicates that farm and parcel turnover is a major driver of intensification processes in the region. Agent management types were also stochastically varied to ascertain the impact that different decision making actors had for the model outcomes. High, standard-deviation between the different model runs for the total area of landscape and nature elements shows that farm types are influential as a driver of landscapes processes.

*Scenario simulation experiments*

The scenarios have been developed to represent different policy actions proposed for the year 2013 (7 model steps). Both the AMIS and BTS scenarios simulate political and economic ruptures that result in local changes to management practices, farm cessation and expansion. It is assumed that alteration in farming incomes, environmental regulations and rural development schemes will induce these changes. Figure i provides the key input data for the different scenario parameters and their link to proposed policy reforms.

|  | **AMIS (market liberalisation)** | **BTS (Sustainable)** |
| --- | --- | --- |
| *Farm cessation*  *(expected overall percentage of agents that stop farming between 2005 and 2020)* | A cessation rate of 0.04% is applied for each turn | A cessation rate of 0.029% is applied each turn |
| *Percentage of agents that stop farming due to EU policy changes (rupture)* | Dairy farmers 15% likely to stop | Dairy farmers 10% likely to stop |
| *Incentives for multifunctional agriculture and small farms* | No, Diversifier 50% more likely to stop in agricultural development zones | Diversifiers 10 % less likely to stop;  Located in national landscape 50 % more likely to continue |
| *Immigration* | Yes, with small farms (>10 DSU) surrounded by more than 10% nature or landscape elements | Yes, with small farms (>10 DSU) surrounded by more than 10% nature or landscape elements |
| *Farming expansion* | Yes, intensive farmer are more likely to expand by a factor of 10%;  This increases to 50% in agricultural expansion zones | Yes, diversified farmer chances expand by 50% if they located in nature and landscape protection areas due to ‘green’ subsidies |
| *Land abandonment* | Low production fields and fields with numerous zoning restrictions for landscape and nature protection | Some nature protection zones purchased by nature organisations |
| *Protection influence of policies related to hedgerows and tree lines* | Landscapes cannot be altered in habitat reserves. Subsides in special landscape zones | Restrictions from cutting elements in landscape protection zones, habitat and corridor areas. |

*Stakeholder formulated policy action simulation experiments*

Three local interventions that were suggested by the mind-mapping groups were added to the model for evaluation. Re-zoning farm management types to appropriate environmental locations was achieved by restricting intensive expansionist farmers from expanding or bequeathing their farms in landscape protection areas, nature corridors (habitat directive) and cultural landscapes. Instead these actors are required to sell their parcels to multifunctional famers, rural residents not primarily engaged in farming or nature organisation. Cooperation and increased tourism were tested together. The distribution of multifunctional farmers throughout the case study area is assumed to allow for cooperation between different farm types if there is a tourism demand. Increased probability for cooperation and tourism demand resulted in increased adoption of multifunctional techniques. A programme to attract in-migration was simulated through increasing demand for smaller rural residencies and decreasing requirements for aesthetically pleasing landscapes around this housing (nature and cultural). This was done by 1) increasing the probability for the purchase of small farms incrementally; and 2) by reducing immigrants’ requirements for nature and landscape elements around the available homestead (10% within a 2 km neighbourhood (circular).
